# Electrical Stimulation Mapping of Brain Function: A Comparison of Subdural Electrodes and Stereo-EEG

**DOI:** 10.3389/fnhum.2020.611291

**Published:** 2020-12-07

**Authors:** Krista M. Grande, Sarah K. Z. Ihnen, Ravindra Arya

**Affiliations:** ^1^Division of Neurology, Comprehensive Epilepsy Center, Cincinnati Children’s Hospital Medical Center, Cincinnati, OH, United States; ^2^Department of Pediatrics, University of Cincinnati College of Medicine, Cincinnati, OH, United States

**Keywords:** functional brain mapping, electrical cortical stimulation, intracranial EEG, epilepsy surgery, drug-resistant epilepsy (DRE)

## Abstract

Despite technological and interpretative advances, the non-invasive modalities used for pre-surgical evaluation of patients with drug-resistant epilepsy (DRE), fail to generate a concordant anatomo-electroclinical hypothesis for the location of the seizure onset zone in many patients. This requires chronic monitoring with intracranial electroencephalography (EEG), which facilitates better localization of the seizure onset zone, and allows evaluation of the functional significance of cortical regions-of-interest by electrical stimulation mapping (ESM). There are two principal modalities for intracranial EEG, namely subdural electrodes and stereotactic depth electrodes (stereo-EEG). Although ESM is considered the gold standard for functional mapping with subdural electrodes, there have been concerns about its utility with stereo-EEG. This is mainly because subdural electrodes allow contiguous sampling of the dorsolateral convexity of cerebral hemispheres, and permit delineation of the extent of eloquent functional areas on the cortical surface. Stereo-EEG, while having relatively sparse sampling on the cortical surface, offers the ability to access the depth of sulci, mesial and basal surfaces of cerebral hemispheres, and deep structures such as the insula, which are largely inaccessible to subdural electrodes. As stereo-EEG is increasingly the preferred modality for intracranial monitoring, we find it opportune to summarize the literature for ESM with stereo-EEG in this narrative review. Emerging evidence shows that ESM for defining functional neuroanatomy is feasible with stereo-EEG, but probably requires a different approach for interpretation and clinical decision making compared to ESM with subdural electrodes. We have also compared ESM with stereo-EEG and subdural electrodes, for current thresholds required to evoke desired functional responses vs. unwanted after-discharges. In this regard, there is preliminary evidence that ESM with stereo-EEG may be safer than ESM with subdural grids. Finally, we have highlighted important unanswered clinical and scientific questions for ESM with stereo-EEG in the hope to encourage future research and collaborative efforts.

## Introduction

A behavioral response to direct electrical stimulation of the human brain was first reported in 1874 from Cincinnati when Bartholow stimulated visible brain tissue in a 30-years-old woman whose parietal bone was eroded by a scalp epithelioma (Bartholow, 1874). He most likely stimulated the left supplementary sensorimotor area, and observed: “…arm was thrown out, the fingers extended, and the leg was projected forward. The muscles of the neck were thrown into action, and the head was strongly defected to the right”. Subsequent pioneering work of Penfield, Ojemann, and others, generated novel information about functional neuroanatomy and established electrical stimulation mapping (ESM) as the gold-standard for pre-surgical localization of eloquent cortical areas (Penfield and Boldrey, 1937; Penfield and Jasper, 1954; Whitaker and Ojemann, 1977; Ojemann and Mateer, 1979; Jayakar et al., 2014). Although early ESM studies allowed some neuroanatomic generalizations based on convergence with lesion data, significant inter-individual variability in the location and extent of eloquent cortical regions was also realized (Ojemann, 1979; Ojemann et al., 2003). This variability in functional anatomy is even more relevant in pediatric patients and those with developmental neuropathology, which may be associated with structural distortion and/or altered plasticity of the functional neuronal networks (Duchowny et al., 1996). Therefore, ESM is necessary for accurate pre-surgical localization of functional areas on an individual basis.

Functional ESM is especially important for patients with drug-resistant epilepsy (DRE) who are being evaluated for neurosurgical treatment. The overarching goal of epilepsy surgery is to ensure long-term seizure freedom and avoid or minimize postoperative neurological deficits. Thus, in preparation for epilepsy surgery, a multi-modal approach is used to develop a patient-specific hypothesis for the location of the seizure onset zone and determine the functional significance of adjacent cortical regions, initially by using several non-invasive tests. However, these non-invasive modalities may not always converge on a single location of the seizure-onset zone and have a limited spatial resolution for defining functional neuroanatomy. Therefore, chronic intracranial electroencephalography (EEG) is required in several DRE patients and offers superior sensitivity for localization of seizure-onset zone mainly because of a higher signal-to-noise ratio compared to scalp EEG (Jayakar et al., 2016). Intracranial EEG also allows the ability to perform ESM by applying small amounts of electrical currents to the same recording arrays and observing behavioral responses. This facilitates the characterization of the functional anatomy of the cortical regions-of-interest, using the principle discovered by Bartholow that electrical brain stimulation may elicit consistent and observable behavioral responses (Table 1).

**Table 1:** Selected landmarks in the history of electrical stimulation mapping of brain function with intracranial electroencephalography (EEG).

Year(s)	Investigator	Significance
1809	Luigi Rolando	Used a voltaic pile and bimetallic electrodes to stimulate the cortex of live animals to consistently produce limb movement.
1848	Gustav Fritsch and Eduard Hitzig	Applied electricity to the exposed cerebral cortex of awake dogs to demonstrate the function of the motor strip.
1874	Roberts Bartholow	Applied electrical stimulation to the human cortex and observed a unique pattern of movements.
1876	David Ferrier	Stimulated the cortex of dogs and monkeys and created a map of functions across the cortex.
1888	Victor Horsely	First to use electrocortical stimulation intraoperatively for localization of the seizure onset zone.
1909	Harvey Cushing	Performed the first awake craniotomy.
1937	Wilder Penfield and Edwin Boldrey	Described the cortical sensorimotor homunculi.
1950s	Wilder Penfield and Herbert Jasper	Pioneered electrocorticography recording with electrical stimulation mapping as part of the “Montreal Procedure” for surgical treatment of epilepsy.
1950s	Robert Hayne and Russell Meyers	Published the first report on stereotactically implanted EEG electrodes in humans with epilepsy.
1950s	Jean Talairachand Jean Bancaud	Developed SEEG and conceptualized the “epileptogenic zone”.
1980s	George Ojemann	Showed importance of individual functional mapping for predicting post-operative function.

*SEEG, stereotactic electroencephalography. This list is not exhaustive*.

**Figure 1:**
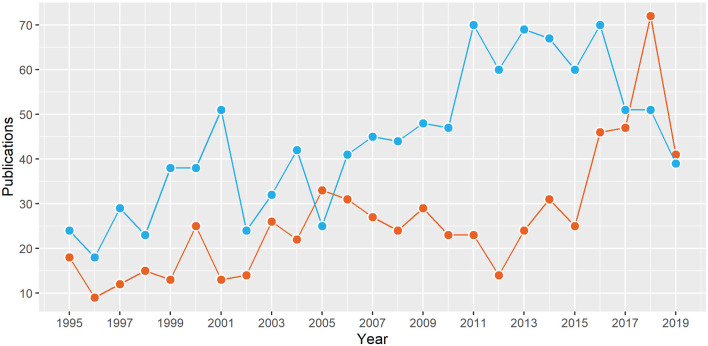
The number of publications from North America on stereotactic electroencephalography (SEEG; red) and subdural electrodes (blue) in the last 25 years. Data from structured PubMed queries (“Electroencephalography”[Mesh]) and “Stereotaxic Techniques”[Mesh] (“Electroencephalography”[Mesh] and “subdural”).

There are two principal modalities for intracranial EEG including subdural electrodes (SDE) and stereo-EEG (SEEG). Although SEEG was developed in the 1960s by Bancaud, Talairach, and others, it has made a resurgence in the last decade in the United States (Figure 1), probably due to advances in neuroimaging and robotics resulting in safer and more precise electrode implantation. A recent Medicare/Medicaid study showed over 1.5 times increase in the use of SEEG as the preferred intracranial modality from 2000 to 2016 (Abou-Al-Shaar et al., 2018). SEEG has been shown to be safer than SDE, with the overall incidence of surgical complications being 0.9–1.7% with SEEG compared to 1.5–4.8% with SDE in large meta-analyses (Arya et al., 2013; Mullin et al., 2016). Also, there is emerging evidence for equivalent seizure outcomes after epilepsy surgery planned with SEEG or SDE (Young et al., 2018; Tandon et al., 2019).

**Figure 2:**
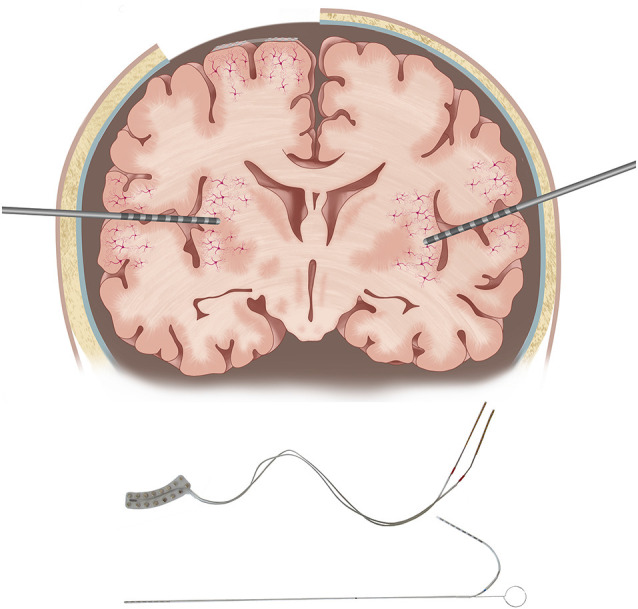
Some important differences between subdural electrodes (SDE) and SEEG electrodes. SDE requires a craniotomy for implantation, while SEEG electrodes are implanted through burr holes. SDE cover the crowns of gyri nearly perpendicular to the dendrites of pyramidal cells, while SEEG electrodes pass at various angles concerning pyramidal cells, through both gray and white matter. Illustrations of an SDE grid and an SEEG electrode are provided in the lower panel.

However, compared to SDE which allows contiguous sampling from the dorsolateral convexity of the cerebral hemispheres, SEEG lacks cortical surface contiguity but allows sampling from deeper cortices, such as insula or depth of sulci, and medial and basal surfaces of cerebral hemispheres, which have minimal or no access with SDE (Figure 2). Given the increasing use of SEEG and concerns about the ability to perform functional mapping due to relatively sparse sampling on the cerebral cortical surface, there is a need to consolidate available information on ESM with SEEG to compare and contrast with SDE ESM (Isnard et al., 2018). Furthermore, although ESM has been performed both intra- and extra-operatively, challenges with awake craniotomy particularly for language testing which requires patient cooperation, severely limit the use of intra-operative ESM in pediatric practice. Therefore, this narrative review will attempt to summarize the evidence for extra-operative ESM with SEEG, compare ESM with SEEG and SDE with a focus on pediatric epilepsy surgery, and will highlight knowledge gaps and potential avenues for future research.

## Procedure

ESM consists of passing small currents through surgically implanted intracranial electrodes and recording behavioral and electrographic responses. Extra-operative ESM is a time and resource-intensive procedure, requiring one or more sessions of several hours each, sometimes spread over multiple days.

### Pre-medication

Some providers choose to administer an anti-seizure medication before ESM to decrease the risks of after-discharges (AD’s) and iatrogenic seizures. However, there is no consensus on this practice, with scant data on its safety and effectiveness. A retrospective pediatric study found that the incidence of ESM-induced seizures was 23% in 40 patients pre-medicated with fosphenytoin, compared to 43% in 82 non-pre-medicated patients (Arya et al., 2018). However, the current threshold for eliciting language responses in the temporal lobe was increased. We could not find any similar study on pre-medication before SEEG ESM, perhaps because eliciting a habitual seizure is sometimes the desired endpoint of SEEG ESM (see later). At our center, we do not routinely pre-medicate patients before SEEG ESM, but have a rescue medication available during the procedure.

### Stimulation Settings

Despite being the gold-standard for pre-surgical localization of cortical functions, ESM remains insufficiently standardized, with heterogeneity in stimulation protocols among different centers (Hamberger et al., 2014). For extra-operative ESM, reasonable settings include pulse widths of 0.2–0.3 ms (range 0.1–1 ms), frequencies of 50 or 60 Hz (1–100 Hz), train durations of 3–5 s (2–10 s), and current amplitudes of 1–20 mA. In terms of current strength, a common practice is to start at 0.5–2 mA and increase by 0.5–1 mA, until a functional response, evolving AD’s, or a seizure is observed, or until the instrument limit is reached. Because of differences in the response properties of various brain regions and different patients, which cannot necessarily be predicted based on clinical factors (Corley et al., 2017), stimulation at each target site should begin at a low current intensity and be optimized. The train duration is dependent on the task. Shorter durations are commonly used for motor mapping, while longer durations are preferred for language mapping to allow sufficient time for the patient to undergo multiple trials of the language task.

The choice of pulse frequency often depends on the need to avoid interaction with the frequency of the electrical mains and is either 50 Hz (in the US) or 60 Hz (in Europe). However, there is some evidence that stimulation of SDE at lower frequencies may result in reduced incidence of AD’s (Zangaladze et al., 2008). In pediatric patients, higher current densities attained using higher current strengths and/or wider pulse widths may be necessary to obtain responses, particularly in children with malformations of cortical development (Chitoku et al., 2001; Sala et al., 2002). Higher functional thresholds in younger children may also be partly explained by a relatively greater proportion of small unmyelinated fibers (Jayakar et al., 1992). Protocols that alternate increases in current intensity with increases in pulse width, called *dual-increment paradigms*, may increase the likelihood of achieving sufficient stimulation, and therefore improve the sensitivity of ESM in the pediatric population (Jayakar et al., 1992).

Intracranial electrodes, whether SDE or SEEG, can be stimulated in a bipolar or unipolar manner. Bipolar stimulation entails the application of recurrent trains of alternating polarity to pairs of often contiguous electrode contacts, whereas unipolar stimulation involves testing a single electrode contact in comparison to a distant reference electrode. Given other settings being constant, the brain volume being electrically stimulated with bipolar stimulation is a function of the distance between the two contacts. Therefore, higher local current densities can be achieved for a given current strength with bipolar stimulation compared to unipolar stimulation (Nathan et al., 1993). We always stimulate in a bipolar fashion.

With SEEG, the stimulation settings are fairly similar to those used with SDE, with two important differences. First, although the maximum current used with SDE ESM has varied from 10 to 20 mA, it has been lower (3–8 mA) with SEEG ESM (Trebuchon and Chauvel, 2016; Britton, 2018; Arya et al., 2019, 2020; Cuisenier et al., 2020). The use of lower maximum current with SEEG ESM is based on calculations extrapolated from the estimates of safe current strengths for SDE ESM, and the desire to maintain similar charge density with SEEG and SDE ESM (Shannon, 1992; Britton, 2018). It is estimated that 8 mA stimulation with a 200 μs pulse will result in charge densities of 12.7 μC/cm^2^ with SDE compared to 31.8 μC/cm^2^ with SEEG electrodes (Britton, 2018). However, the maximum current strength for SEEG ESM remains arbitrary, and not based on rigorous clinical or biophysical evidence. Secondly, it is common in SEEG practice to perform low frequency (1 Hz) stimulation with wider pulse durations (0.5 ms) and train duration (up to 30 s; Cossu et al., 2006; Trebuchon and Chauvel, 2016; Britton, 2018). There is a difference of opinion in the literature if low-frequency stimulation with SEEG should be performed to elicit functional responses or to reproduce habitual seizures (Cossu et al., 2006; Cuisenier et al., 2020). In our experience, low-frequency stimulation can only rarely elicit consistent functional responses. However, we found that 1 Hz stimulation was able to reproduce habitual seizures or auras in 4/6 patients. On the other hand, although 50 Hz stimulation resulted in seizures in 5/15 patients, habitual semiology was observed in only 2/5 patients (Arya et al., 2019, 2020). Therefore, at our center, we prefer 50 Hz stimulation for functional mapping, and 1 Hz stimulation to attempt to reproduce habitual seizures (Table 2).

**Table 2:** Suggested protocol for electrical stimulation mapping with stereo-EEG based on practice at the Cincinnati Children’s Hospital.

Setting	High-frequency stimulation	Low-frequency stimulation
Pulse frequency	50 Hz	1 Hz
Pulse duration	200–300 μs	≤500 μs
Train duration	5 s for language mapping	≤30 s
	2–3 s for motor mapping
Current strength	1–8 mA	1–8 mA
Suggested use	Functional mapping	Seizure induction

*This table was modified and reproduced with permission from Arya et al. (2019)*.

### Clinical Suggestions

Before starting ESM, it is desirable to plan the sequence of stimulation of different electrodes based on the localization of the seizure-onset zone, and presumptive locations of functional areas (either anatomical or obtained from non-invasive modalities). The guiding principle is that electrode(s) lying in the seizure-onset zone should be stimulated last, and only if it is considered essential to stimulate within the ictal core. With SEEG, we always stimulate from deep to superficial contacts, on individual electrodes. This is probably safer due to the lower risk of seizures on white matter stimulation and obviates the concern that deeper contacts may be refractory after stimulation of the superficial contacts in the cortex, because of preferential orthodromic conduction of the stimulation impulse (Trebuchon and Chauvel, 2016). The issue of bipolar stimulation of electrode contacts which may be close to each other in 3D, but lie on different SEEG electrodes, is currently unresolved in the literature.

Even with SEEG, it is preferable to stimulate outwards from the presumptive functional areas. For example, it is preferable to first stimulate the electrode(s) lying in the pre-central gyrus when trying to map the primary motor cortex and then move peripherally towards other electrodes to define the boundaries. This is less intuitive with SEEG compared to SDE. For language ESM, we prefer to stimulate the presumptive frontal language sites first, and then the posterior language regions in temporal and parietal lobes, based on some evidence for directional connectivity from temporal to frontal language regions in the perisylvian language network (Matsumoto et al., 2004).

## Biophysics and Neurophysiology of Esm

The pathway from electrical stimulation of the brain tissue up to the observed behavioral response perhaps has two components. The first stage includes conduction of the stimulation current through the brain tissue and the second stage includes the interaction of this propagated current with different cellular elements to perturb their steady-state. This perturbation in the cellular microenvironment likely results in the observed behavioral response. Both of these stages are extremely complex, and our understanding of the involved processes and variables is currently inadequate to evolve a comprehensive biophysical model for ESM. The core assumption of ESM is that an electric current applied to a small, targeted region of cortex will critically alter local network physiology, resulting in an observable and preferably reproducible response, both within and between patients. In general, an applied current is believed to produce a complex summation effect that depends on multiple factors including, but not limited to, stimulation parameters, electrode type, brain region, lesion type, and patient’s age. Let us consider some of these variables in the following paragraphs.

Intracranial electrodes are usually made from a platinum-iridium alloy or stainless steel, the former being compatible with 1.5T magnetic resonance imaging (MRI) scanners, and in some cases, even with 3T scanners. SDE consists of 3–5 mm metal discs, having 1–2 mm exposed surfaces, embedded in flexible, biocompatible sheets of polyurethane or Silastic, with 5–10 mm center-to-center distance. In comparison, SEEG electrode contacts are cylinders with 0.8–1.2 mm diameter, 1–3 mm length, and 5–10 mm inter-contact distance. While SDE rest on the cortical surface above pia, SEEG electrodes are implanted at an angle to the cortex along a carefully planned trajectory. Thus, it is possible with SEEG ESM that one contact of the stimulated pair may lie in gray matter and another in the white matter. Therefore, the geometry of current spread during SEEG ESM will have to incorporate differences in electrical properties of gray and white matter (Kombos and Suss, 2009; Koessler et al., 2017).

The electric charge delivered by the current pulse is the product of current strength and pulse width. Charge density, in turn, is the electric charge divided by the surface area of the target. For the typical diameter of an SDE disc of 2.5 mm, the contact area is 4.9 mm^2^. Further, for a typical diameter of 0.8 mm and length of 2 mm, the contact area of an SEEG cylinder is 5 mm^2^. Hence, while the contact surfaces of SDE and SEEG electrodes are numerically similar, the charge density at identical settings may be different. In homogeneous media, charge density decreases as the square of the distance from the dipole source. However, as discussed above, SEEG may be susceptible to heterogeneity in electrical properties of the surrounding tissues, especially at the gray-white matter junction. Additionally, in the case of SDE, up to 87% of the current may be shunted by the cerebrospinal fluid, thereby decreasing the effectively delivered current (Nathan et al., 1993). This perhaps justifies using lower current strengths for SEEG ESM compared to SDE ESM.

Another consideration about the spread of applied current is whether it is limited to regional volume conduction or does it propagate through axonal connections. Data suggest that both types of spread may exist. In both monkeys and humans, clinically relevant electrical stimulation has been shown to produce consistent local signal changes on optical imaging, dependent on current intensity and duration, with rapid drop-off as a function of the distance from the electrode tip (Haglund et al., 1992, 1993). In primate models with a recording of functional MRI blood-oxygen-level-dependent response paired to electrical micro-stimulation, the distant trans-synaptic spread of induced current after stimulation of V1 and frontal eye field has been demonstrated (Tolias et al., 2005). In clinical studies, the occurrence of AD’s remote from the site of stimulation, and recording of cortico-cortical evoked potentials (CCEPs) provide evidence for preferential propagation of brain electrical activity (not necessarily the applied current) along functional pathways interconnecting multiple brain regions (Matsumoto et al., 2004, 2012; Afif et al., 2010; Oya et al., 2017; Mălîia et al., 2018; Oane et al., 2020). When functional responses are seen during stimulations associated with distant CCEPs, the localization of the evoked function to the stimulated cortex vs. the remote site with the CCEP remains a dilemma (Afif et al., 2010; Mălîia et al., 2018; Oane et al., 2020). However, there is some evidence for functional responses evoked by activation of remote regions, with the stimulation site perhaps serving as an input into the larger functional network (David et al., 2010). Differences in ESM-induced power modulations in high-frequency (70–150 Hz) SEEG spectra at remote sites, during ESM trials with/without speech/language responses provide support for network effects of local stimulation, and supplement the CCEPs data (Perrone-Bertolotti et al., 2020). Therefore, the functional consequences of transient local current delivery to brain tissue during ESM may be relatively widespread. This is particularly relevant with SEEG because it allows direct stimulation of white matter containing axonal pathways (Sarubbo et al., 2020).

The functional consequences of electrical stimulation of a brain area can include physiologic excitation (functional activation), pathologic excitation (AD’s and seizures), and inhibition (negative function). This has some regional specificity, for example, a majority of stimulations in the primary motor cortex result in activation-type behavior, while those in perisylvian language cortices are primarily inhibitory and are revealed when the patient is actively engaged in a linguistic task. There is evidence that stimulation at the same site can evoke both positive and negative responses (Borchers et al., 2012). The larger repertoire of positive responses reported in the literature may reflect the relative ease of eliciting positive responses rather than negative (inhibitory) responses because negative responses can be detected only when the relevant function is engaged during testing. While it is known that axon initial segments and nodes of Ranvier, rather than cell bodies, are the likely sites of direct neuronal activation because of high concentrations of sodium channels resulting in higher electrical excitability (Rattay, 1999); it remains undetermined if regional differences in cell types or neurotransmitter milieu are responsible for variation in the functional responses. How the interaction of the propagated current with cellular elements translates into the diversity of responses, remains unknown at present.

## Safety Considerations with Esm

In this section, we discuss some of the risks associated with ESM. The risks involved in pre-requisite implantation of SDE or SEEG electrodes are beyond the scope of this article and have been reviewed elsewhere (Arya et al., 2013; Mullin et al., 2016).

### ESM-Induced Seizures

The most consequential adverse event during ESM is the occurrence of an unwanted seizure. ESM-induced seizures are frightening for the patient and the family and may interrupt functional mapping for several hours. In a study including 122 children undergoing SDE ESM (maximum current 15 mA), seizures were triggered in 35% of patients, with secondary generalization in 12% of seizures (Aungaroon et al., 2017). Urgent administration of an anti-seizure medication was needed in 37% of these seizures. This study also showed that current thresholds for the occurrence of seizures and AD’s were significantly associated across the age span and for different lobes of the brain, leading the authors to caution against the continuation of ESM when frequent or evolving AD’s are observed. This finding is consistent with an earlier observation that AD’s may be followed by seizures even in the cortex which does not produce spontaneous seizures (Blume et al., 2004). To compare, the incidence of iatrogenic seizures has varied from 13% to 30% in patients undergoing high-frequency SEEG ESM (Arya et al., 2019, 2020).

Whether ESM-induced seizures can be used to localize the onset of habitual seizures is currently unclear (Kovac et al., 2016). It is common to attempt to reproduce the habitual semiology with SEEG ESM. In a study including 103 SEEG patients, better seizure outcomes were associated with the inclusion of a higher proportion of sites with ESM-induced seizures in the resection (63% vs. 33% electrodes; Oderiz et al., 2019). At our center, we attempt stimulation of habitual seizures, only in patients who have not had a spontaneous seizure usually by the second week of monitoring.

### After-Discharges

AD’s are rhythmic runs of spikes and/or sharp waves that are seen after the electrical stimulation has ceased. The incidence of AD’s has been well documented for ESM with SDE. In a study of 29 patients, aged 6–39 years, 12% of stimulations elicited AD’s (Blume et al., 2004). Importantly, 65% of AD’s involved more than just stimulated electrodes. In another study of 20 adults, 14% of stimulations were associated with AD’s including even those outside the irritative zone (Gollwitzer et al., 2018). In the large pediatric study referred to above, AD’s were seen in 77% of patients (Aungaroon et al., 2017). However, the incidence of AD’s with SEEG ESM has been sparsely reported. Our experience suggests it is probably similar to that with SDE ESM and occurs with 32%–43% of stimulations (Arya et al., 2019, 2020).

Whether stimulation settings used for ESM impact the incidence of AD’s remains unclear. One SDE ESM study showed that the occurrence of AD’s at a given site may be related to prior stimulation at the same site (Lee et al., 2010). This study found that repeat stimulation of the same SDE pair produced AD’s in 19% of trials compared to 5% of trials when a different electrode pair was stimulated. If a trial showed AD’s, the incidence of AD’s on repeat stimulation of the same electrode pair was 46%, compared to 13% if the previous stimulation at the same site was not associated with AD’s. The probability of occurrence of AD’s increased with stimulus duration and decreased with inter-trial interval, leading the authors to suggest waiting for 1 min before repeating stimulation particularly at a site showing AD’s. However, this may further prolong the already lengthy procedure of ESM. In a case report, higher frequency (100 Hz vs. 50 Hz) and longer pulse width (1 ms vs. 0.2 ms) were more likely to cause AD’s (Motamedi et al., 2007). At present, no consistent patient-specific variables that determine the incidence of AD’s have been reported (Aungaroon et al., 2017; Corley et al., 2017).

There is further heterogeneity among studies regarding determinants of threshold currents that produce AD’s. At a group level, a decrease in AD thresholds with age has been documented (see later; Chitoku et al., 2003; Zea Vera et al., 2017), which has been attributed to a higher prevalence of cortical malformations in children (Chitoku et al., 2003), shorter duration of epilepsy (Guojun et al., 2014), and a higher proportion of unmyelinated axons having higher rheobase compared to myelinated axons (Jayakar et al., 1992). However, in addition to inter-person variability, AD thresholds have been shown to vary according to brain region and from day-to-day in the same region (Corley et al., 2017). In a series of 21 patients with SDE, AD thresholds varied from 2 to 15 mA over the tested cortex even between adjacent electrodes (Lesser et al., 1984a), however, in another series of 11 patients, aged 14–47 years, no significant inter-lobar differences in AD thresholds were reported (Suzuki et al., 2018).

The occurrence of AD’s can compromise the safety and neurophysiologic validity of ESM. Whether an associated functional response is attributable to stimulation or AD’s becomes difficult to distinguish. Particularly, in the case of AD’s remote from the site of stimulation, localization of any observed behavioral response to the stimulated cortical site vs. the region showing AD’s, remains unclear.

### Relationship Between AD and Functional Thresholds

An important consideration in designing an optimal ESM strategy is to compare threshold currents required for producing desired functional responses and those resulting in unwanted AD’s. This is particularly relevant in younger children, where functional response thresholds may be higher than older children and adults (Chitoku et al., 2001, 2003). An SDE ESM study including 20 children found AD thresholds to be lower than functional thresholds in children aged 5-years and younger (Jayakar and Lesser, 2008). This was substantiated by a larger (*n* = 122) SDE study that showed language response thresholds to be above AD thresholds throughout the included age range (1–26 years), while motor response thresholds were higher than AD thresholds below 8-years of age (Zea Vera et al., 2017). Therefore, at the group level, the current strengths required to produce functional responses incur a significant risk of producing AD’s. Given the association between the occurrence of AD’s and ESM-induced seizures, as well as their current thresholds, this poses a significant risk with SDE ESM.

**Figure 3:**
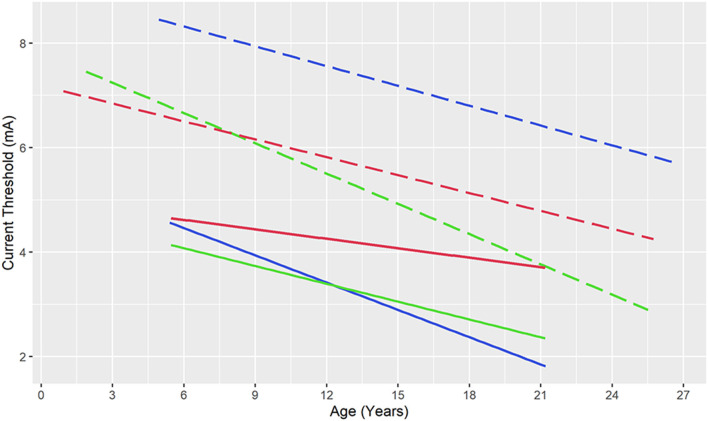
Current thresholds for speech and language responses (blue), motor responses (green), and after-discharges (red) during electrical stimulation mapping with stereotactic electroencephalography (solid lines) and subdural electrodes (dashed lines). Note that after-discharge thresholds are lower than language thresholds (throughout the included age range) and motor thresholds (up to 8-years of age) raising concerns for the safety of subdural electrical stimulation, while functional thresholds remain below after-discharge thresholds for stereotactic electrodes. Ordinary least squares regression lines based on data from Zea Vera et al. (2017) and Arya et al. (2019); Arya et al. (2020).

The preliminary experience with SEEG ESM suggests a different age-relationship between functional and AD thresholds (Figure 3). In 10 patients undergoing language ESM with SEEG, speech/language response thresholds were below AD thresholds throughout the age range (5–21 years; Arya et al., 2019). Similarly, in 15 patients aged 6–21 years, sensorimotor thresholds remained below the AD thresholds (Arya et al., 2020). Therefore, ESM with SEEG may be safer than that with SDE, regarding the risks of ESM-induced AD’s and seizures, because of lower functional thresholds.

### Pain and Tissue Injury

Another potential concern with ESM includes pain on inadvertent stimulation of dura mater including its feeding vasculature, large venous sinuses, or proximal parts of large arteries (Fontaine et al., 2018). The exact incidence of this adverse event with extra-operative ESM is unknown, but is probably low, based on the clinical experience. Because the proportion of electrode contacts with the dura is lower with SEEG, it is expected, but not proven, that nociceptive experiences will be rarer with SEEG compared to SDE.

The application of extrinsic currents during ESM has also raised concerns for tissue injury. Animal studies with prolonged continuous stimulation, sometimes lasting for days, have shown cortical damage, with proposed mechanisms of heat produced by hydrolysis, accumulation of negative charges at the cathode, and generation of metal ions at the anode (Jayakar and Lesser, 2008). However, ESM in humans consists of brief intermittent stimulation with pulses of alternating polarity (biphasic). Therefore, these risks are probably irrelevant in clinical practice. A histological examination of 11 SDE sites in three patients, 1 day after ESM with 50 Hz, 0.3 ms, biphasic pulses at 12.5–15 mA, having estimated charge density of 52–57 μC/cm^2^, did not show any abnormalities attributable to ESM (Gordon et al., 1990). Corresponding data with SEEG ESM is not available at present.

## Functional Responses

There is a wealth of information about experiential and behavioral phenomena associated with intra-operative stimulation and extra-operative ESM with SDE, which has been exhaustively reviewed (Selimbeyoglu and Parvizi, 2010). A systematic review of functional responses seen with SEEG ESM is beyond the scope of this article. Here, we have focused on contrasting the responses seen during SEEG ESM with those seen during SDE ESM. We also limited the discussion to actual responses observed during SEEG ESM and not the semiology components seen during seizure propagation.

### Speech and Language

With SDE ESM, naming errors have been described during stimulation of left inferior frontal gyrus (IFG), left posterior superior temporal gyrus (STG), left anterior middle temporal gyrus (MTG), and bilateral premotor cortex and post-central gyrus (Ojemann and Mateer, 1979; Lesser et al., 1984b, 1994; Lüers et al., 1986; Krauss et al., 1996; Schwartz et al., 1999; Bhatnagar et al., 2000; Corina et al., 2010; Selimbeyoglu and Parvizi, 2010; Suarez et al., 2010). Specific errors of syntactic morphology, word order, and paraphasic errors have been reported with stimulation of left IFG, posterior STG, and anterior MTG, additionally with difficulties in auditory comprehension on stimulation of left posterior STG. Complete anomia has been reported during stimulation at the left IFG, inferior temporal gyrus, and temporoparietal junction.

Similarly, difficulties in naming were seen on stimulation of SEEG electrode contacts located in bilateral STG, transverse temporal gyri, post-central gyrus, and angular gyrus; and left IFG (triangular and opercular parts), MTG, and amygdala (Arya et al., 2019). Interestingly, similar naming deficits were seen on stimulation of white mater SEEG contacts in bilateral temporal and left frontal lobes. Paraphasic errors had an anatomic distribution similar to those seen with SDE ESM but were strictly lateralized to the left cerebral hemisphere. This illustrates an important advantage of ESM with SEEG, which is the ability to study the contribution of deeper structures, such as transverse temporal gyri, essentially inaccessible to SDE, in networks underlying linguistic or other cognitive abilities (see later also).

An important consideration in evaluating ESM speech/language responses is the lack of standardization of task paradigms. In a survey that included 56 epilepsy centers from different countries, a lack of uniformity in what is considered adequate for pre-surgical language mapping was reported (Hamberger et al., 2014). Only half of the epilepsy centers reported testing four main components of language (speech production, comprehension, naming, and reading). Additionally, while over 90% of respondents agreed that non-responses, anomia, and paraphasic errors constituted significant language interruption, the consensus was lacking for the functional significance of hesitations and perseverations. Other areas of discrepancy included interpretation of AD’s and brain regions that should be queried during language ESM. Furthermore, there is evidence for task-specific topography of speech/language function, at least with SDE ESM (Hamberger, 2007). Hence, there is a need to standardize task selection for language ESM, particularly for children and those with limited ability for sustained participation.

### Sensorimotor

SDE ESM was crucial in mapping the somatotopic representation of body parts in the primary sensorimotor cortex located in the pre-and post-central gyri (Penfield and Boldrey, 1937). Additionally, turning of head and eyes, reaching/grasping, and tonic or other postural manifestations in extremities (predominantly contralateral) have been described on stimulation of supplementary sensorimotor area (SSMA) and pre-SSMA (Lim et al., 1994; Kanno et al., 2018). Dysarthria, sometimes severe enough to cause complete speech arrest, is documented on stimulation of bilateral inferior precentral gyrus and left pars opercularis of IFG. Interference with smooth eye movements and generation of saccades is known to be associated with stimulation of frontal eye fields in superior frontal gyri and is rarely seen also on stimulation of right superior parietal lobule (Selimbeyoglu and Parvizi, 2010).

The topography of sensorimotor responses seen with SEEG ESM is largely consistent with that seen with SDE ESM but offers some additional insights. We have reported conjugate gaze deviation on stimulation of superior occipital gyrus and middle cingulate region, suggesting a more elaborate system for control of extra-ocular movements (Arya et al., 2020). We also noted highly specific motor responses such as the extension of the distal inter-phalangeal joint of a single digit, or pronation of proximal radio-ulnar joint, on stimulation of specific sites within pre-central gyrus, which were consistent across patients. This suggests that the cortical representation of certain muscles, most likely intrinsic muscles of the hand, is finer than hitherto realized.

Another possibility with SEEG ESM is to map “negative” motor areas characterized by the arrest of voluntary movements on stimulation. Intra-operative stimulation of white matter underneath the dorsal premotor cortex and SSMA in 18 patients, was found to be associated with cessation of limb movements, interference with bimanual coordination, or speech (Rech et al., 2016). Removal of sites participating in this negative motor network in five patients resulted in post-operative akinesia and mutism, with persistent deficits in fine motor control and bimanual coordination at 3 months, compared to complete recovery in eight patients with preservation of negative motor sites (Rech et al., 2017). Because SEEG allows extra-operative stimulation of white matter, it offers an opportunity to map negative motor sites. However, the continued performance of voluntary movements for such mapping may not be feasible in children and patients with intellectual or motor impairment.

Somatosensory responses were seen with SEEG ESM also have similar localization to those reported with SDE ESM. However, a couple of unique observations are worth mentioning. First, we noted that a majority of somatosensory responses lateralized to the right hemisphere, even for ipsilateral responses (Arya et al., 2020). This was in contrast to the conventional view that well-localized somatic sensations are primarily represented in the contralateral sensorimotor cortex. Some perfusion and lesion studies support our observations and have reported a preeminent role of the right parietal lobe in processing bilateral tactile and thermal stimuli (Peyron et al., 1999; Coghill et al., 2001). Second, we observed that circumscribed tingling, vibrating, or shaking sensations almost always localized to post-central gyrus or underlying parietal white matter, thermal sensations localized to superior parietal lobule, while non-specific sensations had a variable localization. Therefore, we speculated that perhaps the human brain has localization specific to sensory modalities in addition to somatotopic representation. However, these observations require verification by future SEEG ESM studies.

### Mapping the Insula and Cingulate Gyrus

SEEG ESM offers direct access to study functional roles of the insula and cingulate gyrus, which have not been sufficiently mapped with SDE (Figure 2).

Insular stimulations have shown a wide spectrum of responses including special sensory (olfactory, gustatory, and auditory), noxious (suffocation, burning, stinging, and “electric shock”), somatosensory (warmth, paresthesia in various parts of the body), viscerosensory (nausea, epigastric sensation), and vestibular (vertigo; Selimbeyoglu and Parvizi, 2010). Also, psychic or emotional responses (sensation of unreality or out of this world, fear, and anxiety), autonomic phenomena (heart rate changes), and motor responses (automatisms and dysarthria) have been described with insular stimulation, attesting to its integrative role in multiple functional networks (Ostrowsky et al., 2000; Afif et al., 2010; Mazzola et al., 2014, 2019). There is probably some regional specialization within the insula with the posterior insula serving sensory (particularly nociceptive) and vestibular functions, while the anterior insula serving visceral and emotional functions.

There has been limited access to cingulate gyrus for functional mapping with SDE, given the challenges of inserting midline inter-hemispheric strips. However, a wide repertoire of responses on SEEG ESM of the cingulate cortex has been described, which is briefly summarized here. Stimulation of anterior cingulate cortex (anterior to vertical posterior commissure line) has resulted in sensory (epigastric, whole body swaying or rocking sensations), motor (various body parts), anticipatory (intention or urge to move), emotional (laughter, anxiety), speech (arrest), and autonomic (blushing, mydriasis, change in heart rate or respiration, increase in skin conductive response) responses (Talairach et al., 1973; Mangina and Beuzeron-Mangina, 1996; Kahane et al., 2003; Sperli et al., 2006; Mulak et al., 2008). Specifically, motor responses similar to those seen on SSMA stimulation (tonic posturing, reaching, grasping, and eye or head deviation) has resulted in recognition of a cingulate motor area (Chassagnon et al., 2008; Basha et al., 2013; Arya et al., 2020). Stimulation of the posterior cingulate cortex has been associated with similar motor responses and speech arrest, but has additionally shown contralateral upper extremity sensory changes and occasionally visual changes.

### Diagnostic Validity of Localization With SEEG ESM

The diagnostic performance of SEEG ESM for anatomic localization of functional responses has been rigorously evaluated only sparsely. A large adult study (*n* = 209), reported somatic motor responses in 138 (66%) and somatosensory responses in 32 (15%) patients respectively with SEEG ESM (Cossu et al., 2005a). Similarly, sensorimotor responses were reported in 21/35 (60%) of children as well (Cossu et al., 2005b). However, there remains a need to validate the localization of these responses against a reference standard. Ideally, ESM should be evaluated by risk reduction achieved in long-term post-operative neurological or neuropsychological deficits, which requires longitudinal data collection in a large sample.

Therefore, we validated SEEG ESM using a meta-analytic functional MRI framework as reference neuroanatomy, albeit in small samples. Although our comparisons between SEEG ESM performed in individual patients and functional MRI meta-analysis performed in a large heterogeneous dataset are methodologically imperfect, they offer preliminary data for such diagnostic validation. In 10 pediatric patients, we found SEEG ESM (50 Hz, 1–8 mA) to result in speech/language inhibition at 87/304 (29%) electrode contacts. SEEG ESM was a good classifier of anatomic language sites with high specificity (0.87) but limited sensitivity (0.57; Arya et al., 2019). In addition to other methodological differences, this relatively low sensitivity may reflect the wider range of language tasks used to generate the fMRI dataset compared to the single task used for SEEG ESM in our patients. Another recent study including 27 adults (50 Hz, 0.2–3 mA), reported language interference at 85/1914 (4.4%) sites, also with a topography confirming the canonical neuroanatomy (Cuisenier et al., 2020). For sensorimotor mapping, in 15 children, SEEG ESM was noted to localize anatomic sensorimotor parcels with high accuracy (0.80) and high specificity (0.86; Arya et al., 2020).

## Esm and Post-Operative Outcomes

The core purpose of pre-surgical ESM is to establish the functional significance of the seizure-onset zone and establish its anatomical relationship with eloquent cortical areas. This is supposed to help eliminate or minimize post-operative deficits by avoiding eloquent cortical regions during neurosurgery. In case of overlap between seizure-onset zone and eloquent cortex, pre-surgical ESM can help facilitate an informed choice, anticipate potential deficits, and plan for rehabilitation and accommodations.

That epilepsy surgery can affect postoperative motor and cognitive function is well established (Sherman et al., 2011). While epilepsy surgery can improve executive function and memory in some patients, probably by removing the pathological effects of seizures and epileptiform discharges, more frequently it is associated with a decline in verbal memory, naming, attention, and other domains (Lendt et al., 1999; de Koning et al., 2009; Ives-Deliperi and Butler, 2012). The neuropsychological outcomes after epilepsy surgery are driven by multiple factors, including but not limited to the age at onset of seizures, age at the time of surgery, anti-seizure medications, presence and nature of an epileptogenic lesion, location of the seizure-onset zone, its relationship with the eloquent cortices, neurosurgical planning, and most importantly, post-operative seizure outcomes (Skirrow et al., 2011; Puka et al., 2017; Sakpichaisakul et al., 2020). Whether pre-surgical ESM reduces the risk of adverse postoperative outcomes, over and above what may be expected from demographic and clinical information, remains a vexing question. Designing randomized studies to address this question is precluded by ethical principles.

However, the groundwork laid by studies on brain tumor surgery offers some insights. In 40 patients undergoing awake craniotomy for removal of dominant temporal lobe gliomas, the distance of resection margin from the nearest language site was the most important determinant of post-operative language outcomes (Haglund et al., 1994). Authors observed that if this distance was more than 1 cm, significantly fewer permanent language deficits occurred, and proposed it as a safety margin. Another study of 55 patients undergoing resection of tumors located within and adjacent to corticospinal tracts, used tractography-based navigation with real-time guidance from intra-operative ultrasound, and cortical and subcortical motor evoked potentials (Nossek et al., 2011). This study found 87% of their patients recovered without deficits when motor mapping nodes were respected.

The literature on language outcomes after epilepsy surgery, mostly derived from adults undergoing awake craniotomy with intra-operative language ESM, is more heterogeneous. Significantly worse post-operative language function after dominant anterior temporal lobectomy (ATL) was reported in 13/15 patients in one study (Hermann and Wyler, 1988), while no significant deterioration in neuropsychological scores were reported in 18 patients after dominant ATL in another study (Davies et al., 1994). Recently, in 89 patients aged 6–24 years, comprehensive standardized pre- and 1-year post-operative neuropsychological data were examined to evaluate the impact of pre-surgical ESM with SDE (Sakpichaisakul et al., 2020). The multi-domain neuropsychological data were collapsed into three principal component scores representing general cognition, memory, and naming. A significant impact of ESM was seen on all three component scores, in mathematical models adjusted for other clinical variables, with an improvement of 7.5 z-scores in the general cognition score in patients who underwent pre-surgical ESM, vs. a worsening of 8.5 z-scores in those who did not. Only 30% of patients who underwent language ESM experienced a postoperative decrease in general cognition score, compared to 68% of those who did not. Whereas the above study used only a visual naming task for language ESM that is often considered the gold-standard (Hamberger, 2007; Sakpichaisakul et al., 2020), others have emphasized the need for including multiple languages tasks (Wellmer et al., 2009). In a study where language ESM was performed extra-operatively in 12 patients, and intra-operatively in seven patients, all undergoing dominant temporal lobe surgery, all visual naming sites were preserved (Hamberger et al., 2005). However, 6/7 patients with the removal of auditory naming sites experienced a post-operative decline, compared to only 3/12 patients where auditory naming sites were also preserved.

As far as chronic intracranial monitoring of DRE patients is concerned, the above studies are limited to SDE. A large-scale validation of SEEG ESM against post-operative language and neuropsychological outcomes remains an important unmet clinical need. It is also desirable to compare neuropsychological outcomes in cohorts of epilepsy surgery patients evaluated with SEEG vs. SDE, to identify subgroups where one intracranial modality may be preferable over another for ESM.

## Conclusions and Avenues for Future Research

This narrative review attests that SEEG is rapidly becoming the preferred modality for pre-surgical intracranial monitoring given its relative safety compared to SDE and avoidance of craniotomy (Figure 1). Evidence from early studies suggests that seizure outcomes after epilepsy surgery guided respectively by SEEG and SDE are comparable (Young et al., 2018; Tandon et al., 2019). ESM for sensorimotor and speech/language areas is feasible with SEEG, based on the emerging group-level evidence for agreement with reference neuroanatomy (Arya et al., 2019, 2020; Cuisenier et al., 2020). Based on our experience, we suggest 50 Hz ESM for mapping brain function, at pulse widths and train durations similar to those used with SDE ESM, however, using current intensities up to 8 mA (Trebuchon and Chauvel, 2016; Britton, 2018; Arya et al., 2019, 2020). We believe that future studies will further refine this suggested protocol (Table 2). Given that the functional thresholds are lower than AD thresholds for SEEG ESM, compared to the inverse relationship for SDE ESM (Figure 3), it appears that SEEG ESM may be safer compared to SDE ESM, particularly in young children.

However, SEEG ESM requires a different clinical approach for interpretation compared to SDE ESM. While SDE affords surface contiguity and the ability to potentially map the extent of functional areas on the cortical surfaces, SEEG offers the ability to sample from areas typically inaccessible to SDE, including depth of sulci, medial and basal surfaces of cerebral hemispheres, and insula (Figure 2). At our center, after ascertaining the functional significance of electrode contact(s) lying within a particular gyrus using SEEG ESM, we plan surgical resections based on the anatomy of the sulci delimiting that gyrus. SEEG ESM also has the potential to expand our understanding of human functional neuroanatomy. Early studies already show two specific advantages. First, the potential to obtain a more granular map of functional representations, with highly specific sensory or motor responses (Arya et al., 2020). Secondly, the ability to study the functional significance of structures is typically not sampled by SDE (Mazzola et al., 2019; Taussig et al., 2020). SEEG also allows simultaneous bilateral sampling which permits delineating contributions of both cerebral hemispheres in task-related networks, particularly with high-gamma modulation (Forseth et al., 2018; Cuisenier et al., 2020). Translating the methodology of high-gamma modulation mapping from SDE to SEEG is another open research area (Ervin et al., 2020).

Although early studies with SEEG ESM appear encouraging, several clinical and scientific questions remain unanswered. First, there is a need to better understand the physics of current propagation in the brain tissue after bipolar stimulation of SEEG contacts, which may sometimes lie respectively in white matter and gray matter (Koessler et al., 2017). Such biophysical models will then, hopefully, inform the neurophysiology of interaction of these current injections with different cellular elements in the brain tissue to effect electrochemical changes in proximate and distant neuronal assemblies, and ultimately explain the behavioral and experiential phenomena observed with ESM. These biophysical models will also help refine the stimulation protocols by optimizing the local charge density and establishing safety limits for the delivered energy. This is important, because clinical experimentation with different stimulation settings, is largely precluded by ethical principles.

We have mentioned that bipolar stimulation, at least with SDE, may result in the localized current spread. There is some evidence, based on studies of intraoperative motor evoked potentials, that bipolar stimulation may specifically limit current spread perpendicular to the cortical surface (Kombos and Suss, 2009). Therefore, it has been hypothesized that monopolar stimulation may be preferable for excitation of the pyramidal tract. It is likely, but currently unproven, that orthogonally implanted SEEG electrodes may achieve mapping of subcortical tracts at relatively low current settings with bipolar stimulation. Another procedure-related concern is the role of pre-medication before SEEG ESM, including the effect of different medications on cortical excitability and functional thresholds.

To better learn from clinical data, it will be important to develop relatively homogeneous protocols for SEEG ESM. An important consideration will be to develop a modular approach towards the selection of different language and cognitive tasks, particularly for pediatric ESM. Also, it may be desirable to routinely evaluate for interference with voluntary movements during ESM, to map for “negative” motor areas, which may require higher current strengths and sustained patient participation (Kanno et al., 2018). Studies with SDE have shown that neuropsychological deficits do occur after epilepsy surgeries guided by language ESM, particularly in higher-order cognitive domains which may underlie linguistic development and ability, but may not be crucial for cued naming, thus demonstrating the need for multi-paradigm ESM to evaluate different domains (Wellmer et al., 2009; Sakpichaisakul et al., 2020). Although assessment of positive motor responses and patient-reported sensory responses is fairly straightforward, the sensitivity of sensorimotor ESM may be further refined by the use of surface electromyography to detect visually obscure muscle contractions, and by evaluation for negative motor responses by white matter stimulation (Rech et al., 2016, 2017). This is particularly important for SEEG ESM because relatively subtle motor responses are sometimes limited to a single small muscle (Arya et al., 2020). Therefore, it will be worthwhile to develop consensus protocols for SEEG ESM to harmonize data acquisition and enable clinically useful inferences from large samples.

Finally, the most important unmet clinical need is to quantify the risk reduction in adverse post-operative outcomes achieved by pre-surgical SEEG ESM. The purpose of ESM is to help improve the safety of neurosurgical decisions, by offering a safe operative corridor between the seizure-onset zone and eloquent cortical regions. ESM can also help anticipate and prepare for post-operative deficits if there is an overlap between the seizure-onset zone and eloquent functional regions. With SDE, although earlier studies were somewhat discordant (Lendt et al., 1999; de Koning et al., 2009; Skirrow et al., 2011; Puka et al., 2017), a recent large study has demonstrated the relationship between ESM and neuropsychological outcomes (Sakpichaisakul et al., 2020). A multi-center study with sufficient sample size, using valid and comprehensive age-appropriate assessments, both pre-and post-operatively, to establish the predictive value of SEEG ESM for neurological outcomes is imminently needed.

## Author Contributions

All authors participated in literature review, data extraction, and data interpretation. KG and SI drafted the manuscript that was critically reviewed and modified by RA. All authors contributed to the article and approved the submitted version.

## Conflict of Interest

The authors declare that the research was conducted in the absence of any commercial or financial relationships that could be construed as a potential conflict of interest.
